# Risk Factors for Acute Stroke among South Asians Compared to Other Racial/Ethnic Groups

**DOI:** 10.1371/journal.pone.0108901

**Published:** 2014-09-30

**Authors:** Tefera Gezmu, Dona Schneider, Kitaw Demissie, Yong Lin, Martin S. Gizzi

**Affiliations:** 1 Edward J. Bloustein School of Planning and Public Policy, Rutgers the State University of New Jersey, New Brunswick, New Jersey, United States of America; 2 Rutgers-School of Public Health, Department of Epidemiology, Piscataway, New Jersey, United States of America; 3 The New Jersey Neuroscience Institute at the John F. Kennedy Medical Center and Seton Hall University, Edison, New Jersey, United States of America; University of Münster, Germany

## Abstract

**Background:**

Studies of racial/ethnic variations in stroke rarely consider the South Asian population, one of the fastest growing sub-groups in the United States. This study compared risk factors for stroke among South Asians with those for whites, African-Americans, and Hispanics.

**Methods:**

Data on 3290 stroke patients were analyzed to examine risk differences among the four racial/ethnic groups. Data on 3290 patients admitted to a regional stroke center were analyzed to examine risk differences for ischemic stroke (including subtypes of small and large vessel disease) among South Asians, whites, African Americans and Hispanics.

**Results:**

South Asians were younger and had higher rates of diabetes mellitus, blood pressure, and fasting blood glucose levels than other race/ethnicities. Prevalence of diabetic and antiplatelet medication use, as well as the incidence of small-artery occlusion ischemic stroke was also higher among South Asians. South Asians were almost a decade younger and had comparable socioeconomic levels as whites; however, their stroke risk factors were comparable to that of African Americans and Hispanics.

**Discussion:**

Observed differences in stroke may be explained by dietary and life style choices of South Asian-Americans, risk factors that are potentially modifiable. Future population and epidemiologic studies should consider growing ethnic minority groups in the examination of the nature, outcome, and medical care profiles of stroke.

## Introduction

The incidence and death rate from stroke have declined in the United States [1]–[2],while racial/ethnic, socioeconomic, and gender disparities remain. The literature abounds with studies on racial/ethnic disparities in stroke mortality and behavioral risk factors for stroke; however few studies examine the different types of stroke among ethnic minorities and almost none focus on South Asians. According to the US Census, the South Asian population in the United States increased by 43.3% between 2000 and 2010, a proportion more than four times the 9.7% increase for the general population [3]–[4]. In New Jersey alone, the number of people who identified themselves as South Asian increased by 51% (480,276 in 2000 to 725,726 in 2010) [3], [5]. Thus, New Jersey provides an opportunity to address this gap in the stroke disparities literature.

Although White and colleagues investigated the incidence of ischemic stroke subtypes among racial/ethnic groups [6], most other studies of racial/ethnic differences in stroke compare the prevalence of risk factors alone [7]–[10]. As a result, little is known about the differences in the pathogenesis, prognosis, and treatment of ischemic stroke subtypes for different racial/ethnic groups. This study compares the stroke experience of South Asians with that of other racial/ethnic groups in a highly diverse population served by a comprehensive regional stroke center.

## Methods

### Data Source

Data on all acute stroke cases were prospectively collected at the time of admission to the New Jersey Neuroscience Institute at John F. Kennedy (JFK) Medical Center in Edison, New Jersey. All variables related to stroke diagnosis, treatment, and outcomes were abstracted from the medical records of patients by trained personnel using a standardized algorithm and entered into a computerized registry system. These included the Center for Disease Control's Paul Coverdell Stroke Registry variables, and the trial of Org 10172 in Acute Stroke Treatment (TOAST) etiologic classifications elements. Predictive factors for stroke included age, sex, race/ethnicity, obesity, diabetes mellitus, hypertension, cardiac diseases, and lipid profiles. Data on history of cigarette smoking, past stroke or Transient Ischemic Attack (TIA), prior prescription medication use, and other comorbidities were collected, along with clinical and anthropometric measures.

### Study Populations

There were 3,540 acute stroke admissions between 2006 and 2011. After excluding 250 (7.1%) patients who did not meet the racial/ethnic categorization (white, African American, South Asian or Hispanic) and those who were ≤18 years of age, 3,290 acute stroke patients were selected for this analysis. All patients who self-identified India or Pakistan as country of origin were classified as South Asian; as many South Asians self-identify as white; the surnames of the remaining patients were examined to determine race/ethnicity. International medical graduates of South Asian origins that were trained in data abstraction and surname recognition performed surname identification. They categorized patients with surnames common to Bangladesh, Bhutan, India, Nepal, Pakistan, Sri Lanka, and the Maldives as South Asians [11]. This method has been validated by the RAND cooperation and the US Census bureau, among others [12]–[15]. To capture individual level data for patients' education, occupation and income, socioeconomic status (SES), patient zip codes were matched to the US Census of Population (2000) [5]. Only 0.05% of patients lacked zip code information and SES could not be assigned.

### Clinical Outcomes

All patients were evaluated with standard blood tests for fasting glucose, hemoglobin A1C (HbA1c) as well as lipid and coagulation profiles. Computed tomography (CT) scans of the brain were performed at the time of initial evaluation and again in 24 hours. The majority of patients underwent magnetic resonance imaging (MRI) of the brain. Patients had extra-cranial vascular imaging using CT angiography (CTA), and MR angiography (MRA). All patients undergoing CTA or MRA also had intracranial vascular imaging and 2D trans-thoracic echocardiography, and either Holter monitor or a minimum of 24 hours of telemetric cardiac monitoring. Patients with a suspected embolic source underwent trans-esophageal imaging.

Acuity and extent of neurological deficits from stroke were determined according to the NIHSS scoring system [16]–[17]. TOAST classification was performed by stroke-trained neurologists using the criteria published by Adams et al. (1993) [18]. Stroke classifications include large-artery atherosclerosis (LAA), embolism of cardiac origin (CE), small-artery occlusions (SAO), other determined/known cause, and undetermined/unknown causes (including two or more of the above) [18]–[19]. Patients with two or more identified causes of ischemic stroke were classified as undetermined causes of ischemic stroke.

### Vascular Disease Risk Factors

Diagnosis of diabetes was established if a patient was under treatment with an anti-diabetic medication or insulin or had a fasting glucose level ≥140 mg/dL, or a history of type I or II diabetes [20]. Hypertension was established if patients were on antihypertensive therapy or if their systolic/diastolic blood pressures were ≥160/90 mm Hg upon admission [21]. Hypercholesterolemia was defined as total cholesterol (TC) ≥240 mg/dL, or under treatment with cholesterol-reducing medication [22]. Obesity was determined by body mass index (BMI). Cardiac disease was defined as a history of coronary artery disease, myocardial infarction, congestive heart failure, angina, coronary artery bypass graft, angioplasty, atrial fibrillation and other arrhythmias, or any current use of cardiac medications. Smoking was defined if patient smoked at least one cigarette during the year.

### Statistical Analysis

Institutional Review Board approval to evaluate the data was obtained both from the JFK Medical Center and the University of Medicine and Dentistry of New Jersey. Patient informed consent to examine the medical record was not obtained since data abstractions were anonymized/de-identified and included no Patient Health Information (PHI). All variables were summarized by race/ethnicity and reported as mean ± standard deviation (SD) or median and interquartile range as appropriate. Variables were compared among racial/ethnic groups with χ2 or Fisher's exact tests for categorical variables and the Kruskal-Wallis tests for continuous variables, while differences in means between racial/ethnic groups were compared using analysis of variance (ANOVA). Because triglycerides, hemoglobin A1c, fasting blood glucose and BMI values were positively skewed, natural logarithmic transformation was used and results were expressed as geometric means with 95% confidence intervals (CI). Finally, the distributions of ischemic stroke subtypes were examined by race/ethnicity. All analyses were done using SAS statistical software, version 9.2 (SAS Institute Inc., Cary, NC).

## Results

The socio-demographic characteristics of study subjects are detailed in Table 1. Among the 3,290 stroke admissions, 65.5% were white, 18.4% were African American, 8.3% were South Asian and 7.8% were Hispanic. Women accounted for over half of all stroke patients in the cohort; however their proportions were lower among South Asians and Hispanics. There were significant differences in age by race/ethnicity (P<0.0001). The overall mean age was 70 years, with whites the oldest among the racial/ethnic groups (mean = 73 years). South Asians were much younger (mean = 65 years) with only 11% on Medicare as compared to 23% of whites. Socioeconomic status comparison by zip code revealed that South Asians tended to reside in areas with the highest median family income ($78,389). South Asian patients were also more likely to be college graduates as compared to other racial/ethnic groups.

**Table 1:** Characteristics of patients with acute stroke by race/ethnicity.

Variable	South Asians N = 273	Whites N = 2156	African Americans N = 605	Hispanics N = 256	Total N = 3290	P^a^
**Demographics**						
Mean age, years (SD)	65.1 (14.5)	73.0 (14.4)	64.7 (14.5)	63.8 (16.1)	70.0 (15.1)	<0.0001^b^
% Female	40.7	55.0	59.7	46.9	54.0	<0.0001
**Insurance Status, %**						0.9441^c^
Medicare	10.6	23.1	18.0	21.5	21.0	
Medicaid	2.6	0.7	2.1	1.6	1.2	
Medicare and Medicaid	16.5	3.5	8.4	8.6	5.9	
Commercial/VA	25.3	16.6	20.7	16.4	18.0	
Self-Pay/None	22.3	2.8	12.2	16.0	7.2	
HMO	0.0	0.1	0.2	0.0	0.1	
Undetermined	0.8	0.1	0.0	0.0	0.1	
Any Combination Above	21.9	53.1	38.4	35.9	46.5	
**Socioeconomic Status**						
% Unemployed	5.9	40.6	28.2	25.3	4.1	<0.0001
Median Household Income (IQR)	$78,389 (17,446)	$62,964 (11,689)	$50,707 (15,374)	$47,708 (23,174)	$61,091 (18,805)	0.1465^d^
Median Household Size	3.0	3.3	1.8	1.8	2.7	
**% Education Level**						<0.0001
<High School	17.2	16.8	32.0	50.3	23.6	
High School Graduate	14.1	36.2	38.1	30.3	34.1	
College Graduate	68.7	47.0	29.9	19.4	42.3	
**% Below Poverty Line**	6.3	33.0	28.0	32.7	19.5	<0.0001

HMO = Health Maintenance Organization.

VA = Veterans Affairs.

NS = not significant.

SD = standard deviation.

IQR = interquartile range.

a χ^2^ test.

b ANOVA comparing mean age by race/ethnicity.

c Fisher's exact test.

d Kruskal-Wallis test.

Table 2 and 3 detail the distribution of stroke risk factors, history of prescription medication use and clinical measurements related to stroke by race/ethnicity. Hypertension was the most frequent risk factor for stroke among all racial/ethnic groups, ranging from 82% among African Americans to 70% among Hispanics. The prevalence of hypertension was similar between the South Asian and white subjects in this study, at 75.5% and 76.7%, respectively. However, there was a significant difference between South Asians and African Americans, p = 0.0254 (pairwise comparison). Comorbidities such as diabetes mellitus, cardiac disease and past history of stroke/TIA were noteworthy risk burdens for all racial/ethnic categories, Table 2. South Asians had statistically significantly higher prevalence of diabetes mellitus when compared to whites (p<0.0001) and Hispanics (p = 0.0022). South Asians also reported a lower rate of antihypertensive medications use as compared to whites and African Americans but higher than Hispanics; however, only 9.2% of South Asians reported any history of smoking, a well-known stroke risk factor, Table 2.

**Table 2:** Prevalence of risk factors and history of medication use among stroke patients by race/ethnicity.

Variable	South Asian N = 273	White N = 2156	African American N = 605	Hispanic N = 256	P^a^
**Risk Factor %**					
Hypertension	75.5	76.7	82.0	70.0	0.0010
*P* c	*-*	*0.656*	*0.0254*	*0.1528*	
Diabetes mellitus	45.4	30.7	42.2	32.4	<0.0001
*P* c	*-*	*<0.0001*	*0.3648*	*0.0022*	
Cardiac diseases^b^	24.5	36.0	27.9	22.3	<0.0001
*P* c	*-*	*0.0002*	*0.2940*	*0.5368*	
Dyslipidemia	33.5	35.5	30.7	32.0	0.9994
*P* c	*-*	*0.9994*	*0.1600*	*0.3952*	
Smoker	9.2	17.2	15.4	8.2	<0.0001
*P* c	*-*	*0.0007*	*0.0125*	*0.6970*	
Prior stroke or TIA	31.1	33.3	39.8	28.5	0.0028
*P* c	*-*	*0.4734*	*0.0135*	*0.5106*	
Any Combination	49.1	48.8	47.3	45.3	0.4734
*P* c	*-*	*0.9280*	*0.6189*	*0.3852*	
**Prescription Medication %**					
Antihypertensive	68.5	73.2	72.7	61.3	0.0005
*P* c	*-*	*0.1012*	*0.1992*	*0.0840*	
Cholesterol	37.4	44.9	37.2	38.3	0.0007
*P* c	*-*	*0.0175*	*0.9610*	*0.8276*	
Antiplatelet	45.1	44.1	37.5	35.9	0.0037
*P* c	*-*	*0.7559*	*0.0348*	*0.0329*	
Anticoagulant	27.1	36.9	33.1	28.1	0.0007
*P* c	*-*	*0.0015*	*0.0781*	*0.7934*	
Diabetic	41.4	25.1	35.2	27.3	<0.0001
*P* c	*-*	*<0.0001*	*0.0791*	*0.0007*	

NS = Not Significant.

TIA = Transient Ischemic Attack.

LDL = Low Density Lipoprotein.

HDL = High Density Lipoprotein.

a P-value from a χ2 test for the overall race/ethnicity effect.

b Includes atrial fibrillation, prosthetic heart valve, coronary artery disease/prior myocardial infarction, carotid stenosis, peripheral vascular disease and heart failure.

c P-values from chi-square tests for overall race/ethnicity effect followed by pairwise comparison between race/ethnicities. A significant level of 0.05 was used for the overall race/ethnicity comparisons. Bonferroni adjustments created a significance level of 0.05/2 = 0.025 (since South Asians were to each race/ethnicity at a time).

**Table 3:** Clinical measurements at the time of admission among stroke patients by race/ethnicity.

Variable		South Asian N = 273	White N = 2156	African American N = 605	Hispanic N = 256	P^a^
**Mean clinical measurements (SD) or (95% CI)** b						
Blood pressure (mmHg)	Systolic	156.8 (32.6)	152.7(30.5)	153.8 (33.0)	151.7 (28.2)	<0.1929
	*P* c	-	0.1004	0.1506	0.3719	
	Diastolic	87.4 (21.1)	82.7 (18.7)	86.2 (20.1)	83.0 (17.2)	<0.0001
	*P* c	-	0.0006	0.6604	0.0232	
Total cholesterol (mg/dl)		172.3 (44.9)	171.6 (44.5)	177.5 (47.3)	177.2 (50.1)	0.0397
*P* c		-	0.9891	0.2994	0.4883	
LDL (mg/dl)		105.6 (38.2)	104.2 (38.5)	110.2 (38.8)	111.2 (40.4)	0.0032
*P* c		-	<0.0001	0.2663	0.2575	
HDL (mg/dl)		41.5 (11.9)	43.4 (13.7)	45.1 (15.2)	41.0 (11.3)	0.0003
*P* c		-	0.0818	0.0019	0.9543	
Triglycerides (mg/dl)		115.8	107.4	92.1	112.4	<0.0001
		(108.3–115.8)	(105.1–109.9)	(88.0–96.5)	(108.3–123.7)	
*P* c		-	0.0753	<0.0001	0.8383	
HbA1c (%)		6.9	6.3	6.7	6.5	0.0143
		(6.7–7.1)	(6.2–6.3)	(6.5–6.8)	(6.3–6.7)	
*P* c		-	<0.0001	0.0454	0.0083	
Fasting blood glucose (mg/dl)		132.8	122.4	128.8	130.2	<0.0001
		(126.4–139.6)	(120.7–124.2)	(124.6–133.3)	(123.7–137.2)	
*P* c		-	0.0016	0.4810	0.8358	
Body mass index (kg/m^2^)		25.3	26.9	28.3	27.8	<0.0001
		(24.8–25.9)	(26.6–27.2)	(27.7–28.8)	(27.1–28.5)	
*P* c		-	0.0001	<0.0001	<0.0001	

a Overall p-value from a type 3 tests of fixed effects.

b 95%CI: 95% Confidence Interval.

c P-values for the overall race/ethnicity effect followed by pairwise comparison between race/ethnicities. Dunnett adjustment was applied to correct for pairwise comparisons. Results were considered statistically significant when *p* values were less than 0.05.

Stroke related clinical measurements at the time of admission by race/ethnicity are detailed in Table 3. Although the overall race/ethnicity comparison of systolic blood pressure did not show any race/ethnicity effect, South Asians had a significantly higher mean diastolic blood pressure levels when compared to whites and Hispanics (pairwise comparison, p = 0.0006 and p = 0.0232, respectively). Additionally, South Asians had higher triglycerides levels than African Americans (p<0.0001); South Asians also had significantly higher levels of HbA1c than all other race/ethnicity groups, Table 3. Although, there was no significant difference in the mean TC blood concentrations levels; South Asians had significantly lower circulating HDL levels than African Americans (p = 0.0019; Table 3).

Table 4 compares clinical characteristics, vital signs and lipid profiles of acute stroke patients' by race/ethnicity using categories accepted in the clinical practice, diagnosis and management guidelines for hypertension, diabetes and hypercholesterolemia [21]–[22]. Higher proportion of South Asians had Blood pressure levels>160/100 mmHg when compared to all other racial/ethnic groups, Table 4 (overall race/ethnicity effect p-value = 0.002). About 40% of South Asian patients had fasting blood glucose levels ≥126 mm/dL, while only 33% of whites and 37.5% of African Americans fall in this category (overall race/ethnicity effect p = 0.028), however pairwise comparison showed no statistical significant differences. The distribution of low-density lipoproteins that were considered to be high or very high (where LDL≥160 mg/dL) was lower among South Asians than among African Americans and Hispanics (10.6 and 8.9%, respectively). Moreover, larger proportion of South Asians had lower levels of high-density lipoproteins (where HDL≤40 mg/dL) than all other racial/ethnic groups (overall race/ethnicity effect p<0.0001), a potent indicator of the risk for cardiovascular diseases.

**Table 4:** Clinical characteristics, signs and lipid profiles of stroke patients by race/ethnicity vital.

Variable	South Asian N = 273	White N = 2156	African American N = 605	Hispanic N = 256	P^a^
**Blood Pressure (mmHg)** b					0.0020
≤120/80	35.1	42.2	34.7	40.2	
120–139/80–89	23.1	20.8	22.0	24.6	
140–159/90–99	16.1	16.8	18.0	12.9	
>160/100	19.1	12.2	16.2	13.3	
Missing	6.6	7.9	9.1	9.0	
**Fasting Blood Glucose (mm/dL)** c					0.0280
<126	51.3	55.7	50.0	48.8	
≥126	40.0	33.0	37.5	39.8	
Missing	8.8	11.4	12.6	11.3	
**HbA1c Classifications** c					<0.0001
<6.5%	34.8	44.4	38.0	40.6	
≥6.5%	37.0	17.7	29.0	24.6	
Missing	28.2	37.8	33.1	34.8	
**Body Mass Index (kg/m^2^)** d					<0.0001
≤18.5	2.9	2.2	2.2	1.2	
18.6–24.9	44.0	32.4	24.1	24.2	
25.0–29.9	27.5	31.2	30.4	38.0	
≥30.0	17.0	23.1	30.6	26.6	
Missing	8.8	11.1	12.7	10.2	
**Total Cholesterol (mg/dL)**					0.0171
<200	68.5	64.1	61.0	57.4	
200–239	12.1	12.9	17.2	15.2	
>240	7.0	6.0	7.6	8.2	
Missing	12.5	17.1	14.2	19.1	
**Low Density Lipoprotein (mg/dL)**					0.0202
<129	66.7	65.4	62.6	59.0	
130–159	13.9	11.0	13.4	13.7	
160–189	4.8	5.0	8.4	6.6	
≥190	2.9	2.2	2.2	2.3	
Missing	11.7	16.4	13.4	18.4	
**High Density Lipoprotein (mg/dL)**					0.0001
<40	44.7	36.1	32.1	39.8	
40–59.9	37.0	36.9	41.7	35.9	
≥60	5.2	9.8	12.2	5.1	
Missing	13.1	17.2	14.1	12.5	
**Triglycerides (mg/dL)**					<0.0001
<150	59.3	63.4	72.7	63.6	
150–199	16.5	12.3	8.9	9.0	
200–499	11.4	6.8	4.5	10.6	
≥500	0.7	0.3	0.2	0.8	
Missing	12.1	17.2	13.7	12.1	

a Kruskal-Wallis test.

b Based on the 7th report of the Joint National Committee on Hypertension (JNC7).

c Criteria for diagnosis of diabetes by the American Diabetes Association, 2010.

d Centers for Disease Control BMI guidelines for weight status.

A comparison of the overall race/ethnicity effect in the incidences of stroke types and ischemic stroke subtypes showed a significant variation, p = 0.0040; however, pairwise comparison of South Asians to the other racial/ethnic groups did not show any statistical effect, Table 5. Ischemic stroke was the most frequent reason for acute stroke admission among this cohort. South Asians had the highest proportion of patients with ischemic stroke (∼50%) when compared to other racial/ethnic groups. The examination of the TOAST etiologic classifications among the 1541 ischemic stroke cases showed that there was an overall race/ethnicity effect in variation of ischemic stroke subtypes (overall race/ethnicity effect p = 0.0013, Table 5). Specifically, South Asian patients had the highest proportion of admissions with SAO diagnosis (29.4%) as compared to other racial/ethnic groups. South Asians also had a statistically significantly higher proportion of patients with LAA subtype diagnosis than whites (pairwise comparison p-value = 0.0123, Table 5). Overall LAA diagnosis was more common among Hispanics 30.5%, followed by South Asians at 26.5%. Only 8.1% South Asians had ischemic stroke subtype diagnosis of cardio embolic etiology as compared to 19.7% of white patients, Table 5.

**Table 5:** Incidence of stroke types^a^ and ischemic stroke sub-types by race/ethnicity.

Variable	South Asian N = 273	White N = 2156	African American N = 605	Hispanic N = 256	Total N = 3290	P^b^
**Clinical Diagnosis, %**						
Ischemic Stroke	49.8	47.0	47.4	41.0	46.8	0.0040 (South Asians vs. Whites p = 0.1843; South Asians vs. AAs p = 0.0866; and South Asians vs. Hispanics p = 0.1502)
Transient Ischemic Attack	30.4	36.3	37.4	34.3	35.8	
Hemorrhagic Stroke	17.6	14.9	13.9	21.5	15.5	
Subarachnoid	4.4	3.3	4.1	8.2	3.9	
Intracerebral	13.2	11.6	9.8	13.3	1.6	
Unspecified	0.7	0.7	1.0	0.4	0.7	
No Stroke Diagnosis	1.5	0.6	0.2	1.6	0.6	
c **Stroke etiology, %**	n = 136	n = 1013	n = 287	n = 105	n = 1541	
Large-artery atherosclerosis	26.5	23.1	20.2	30.5	23.4	0.0013 (South Asians vs. Whites p = 0.0123; South Asians vs. AAs p = 0.0920; and South Asians vs. Hispanics p = 0.1164)
Small-artery occlusions	29.4	22.0	23.0	23.8	23.0	
Cardioembolism	8.1	19.7	11.9	15.2	16.9	
Other	35.3	34.2	43.2	27.7	35.5	
Determined	0.7	2.7	4.5	2.9	2.9	
Undetermined	34.6	31.5	38.7	24.8	32.6	

a On average less than 3% of data was missing. Missing data was largely among Hispanics and ranged from 1.2 to 2.9%.

b P-values from chi-square tests for overall race/ethnicity effect followed by pairwise comparison between race/ethnicities. A significant level of 0.05 was used for the overall race/ethnicity comparisons. Bonferroni adjustments created a significance level of 0.05/2 = 0.025 (since South Asians were to each race/ethnicity at a time).

c Ischemic stroke classification scheme from the Trial of ORG 10172 in Acute Stroke Treatment (TOAST).

## Discussion

Stroke remains a major health issue, particularly among minority and ethnic sub-groups. For example, despite being almost a decade younger than white patients, having more college graduates, and residing in neighborhoods with the highest median income compared to other study subjects, South Asians had clinical risk factors for stroke comparable to those of African Americans and often worse than those for Hispanics. It is well established that hypertension is potent risk factor for stroke. This analysis showed it to be the most prevalent risk factor for all stroke victims, distantly trailed by diabetes mellitus and cardiac diseases. South Asian and white patients in our cohort had higher proportions of subjects with hypertension than Hispanics. Prevalence of smoking among South Asians in this study (which included Asian Indians) was consistent with a 2012 report by the Center for Disease Control and Prevention (CDC) [23] that indicated that only about 12% of Asian Indian survey respondents reported smoking within the past 30 days. As is the case for many US immigrants, South Asians may have lower use or access to primary care services or, even if they are not normally hypertensive, suffer a higher prevalence of blood pressure in the acute phase of stroke. Among our cohort, race/ethnicity accounted for significant variability in HbA1c, fasting blood glucose and blood pressure measurements at the time of admission. South Asians had the highest proportion of patients with these risk factors, while TC and LDL levels were highest among African American and Hispanic patients. One explanation for these findings is that South Asians may be more prone to stress-induced hyperglycemia.

Great care should be taken in interpreting the findings of this study, especially because the diagnosis of hypertension and diagnosis for diabetes based on FBG levels ≥140 mg/dL are often questionable if not performed during a medically quiescent period. However, the prognosis of developing diabetes on HbA1C and FBG levels is generally considered reliable.

White and colleagues reported that blacks are at a higher risk for intracerebral atherosclerosis compared with whites [6]. We found that South Asian and Hispanics patients were equally likely to be diagnosed with the stoke sequelae of this disease as whites or African Americans. Furthermore, we found a high proportion of ischemic stroke distributed to “other undetermined causes” a classification including “two or more causes” of stroke etiologies, a situation common among patients with small vessel disease. It is possible that the high prevalence of ischemic stroke of undetermined causes among the African American patients in our cohort is correlated with a higher incidence of cardiovascular risk factors in the general African American population.

Risk factors for ischemic stroke varied significantly by race/ethnicity among our cohort. There was>25% increased risk for developing SAO among South Asian patients than whites. This can be explained by the substantial age difference between these two groups in that South Asians tended to be a decade or so younger than whites and more likely to suffer from SAO at earlier ages. This competing morbidity perhaps reduced their risk of LAA at older ages. Indeed, SAO was more common among South Asians than any other racial/ethnic group. However, the conclusion regarding these factors among South Asians may not hold true when examining South Asians living in their home countries.

The strength of our study stems from the fact that the data were collected on subjects that were assessed immediately following the onset of stroke symptoms by neurologists and a dedicated stroke team at a comprehensive stroke center. Data gathered in this manner have been reported to have a less than 2% error rate of stroke clinical diagnosis [23]. A possible limitation in the overall interpretations of results here is that the fairly large sample size may precipitate a statistically significant result with no clinical significance. The reader is reminded to exercise value judgment when making any inference based on the result of larger sample sized studies as this one.

## Conclusions

In order to improve stroke outcomes and to achieve the impact goals set by the American Heart Association to reduce stroke death by 20% by 2020, future epidemiologic studies that investigate and identify the most efficient and effective clinical approaches to both prevention and treatment are important. The higher prevalence of known stroke risk factors among South Asian immigrants in this cohort may have been attributable for the larger proportion of ischemic stroke incidence among South Asians as compared to whites, including the increased incidence of small vessel disease”. These findings support the need for genetic and metabolomics studies that may lead to an understanding of the mechanisms of at least one stoke subtype. The further examination of the interaction between race/ethnicity and stroke risk factors can provide data that will enable clinicians to predict subtypes of stroke following an acute stroke admission and to act on that knowledge accordingly.

## References

[1] Fang J, Alderman MH, Tu JV (2001) Trend of Stroke Hospitalization, United States, 1988–1997 Editorial Comment. Stroke 32 (10) 2221–2226. 11588304 10.1161/hs1001.096193

[2] Pathak EB, Sloan MA (2009) Recent Racial/Ethnic Disparities in Stroke Hospitalizations and Outcomes for Young Adults in Florida, 2001–2006. Neuroepidemiology 32 (4) 302–311.. 19287184 10.1159/000208795

[3] U.S. Census Bureau AFF. The Asian Population in the United States. Census 2010 Census Results; Available: http://factfinder2.census.gov/faces/nav/jsf/pages/index.xhtml. Accessed 2010 Dec 20.

[4] U.S. Census Bureau AFF. Race Data. Census 2010 Census Results; Available: http://2010.census.gov/2010census/data/. Accessed 2011 Oct 13.

[5] United States Department of Health and Human Services (US DHHS), Centers for Disease Control and Prevention (CDC), Bridged-race Intercensal Population Estimates and 2000–2008 (Vintage 2008) On-line Database. Available: http://wonder.cdc.gov/bridged-race-v2008.html Accessed 2010 Jun 9.

[6] White H, Boden-Albala B, Wang C, Elkind MSV, Rundek T, et al (2005) Ischemic Stroke Subtype Incidence Among Whites, Blacks, and Hispanics: The Northern Manhattan Study. Circulation 111 (10) 1327–1331.. 15769776 10.1161/01.CIR.0000157736.19739.D0

[7] Boden-Albala B, Sacco RL, Lee H-S, Grahame-Clarke C, Rundek T, et al (2008) Metabolic Syndrome and Ischemic Stroke Risk: Northern Manhattan Study. Stroke 39 (1) 30–35.. 18063821 10.1161/STROKEAHA.107.496588 PMC2677015

[8] Liao Y, Greenlund KJ, Croft JB, Keenan NL, Giles WH (2009) Factors Explaining Excess Stroke Prevalence in the US Stroke Belt. Stroke 40 (10) 3336–3341. 19679841 10.1161/STROKEAHA.109.561688

[9] Sacco RL, Benjamin EJ, Broderick JP, Dyken M, Easton JD, et al (1997) Risk Factors. Stroke 28 (7) 1507–1517.. 9227708 10.1161/01.str.28.7.1507

[10] Sacco RL, Roberts JK, Boden-Albala B, Gu Q, Lin IF, et al (1997) Race-Ethnicity and Determinants of Carotid Atherosclerosis in a Multiethnic Population: The Northern Manhattan Stroke Study. Stroke 28 (5) 929–935.. 9158627 10.1161/01.str.28.5.929

[11] Forbes J, Fisher M. The Cancer Institute of New Jersey and South Asian Total Health Initiative Join Forces. Available: http://rwjms.umdnj.edu/news_publications/news_release/2010_release/CINJ_SATHI_screenings.html Accessed 2010 Dec 20.

[12] Wong EC, Palaniappan LP, Lauderdale DS (2010) Using Name Lists to Infer Asian Racial/Ethnic Subgroups in the Healthcare Setting. Medical Care 48 (6) 540–546.. 20421828 10.1097/MLR.0b013e3181d559e9 PMC3249427

[13] McAlpine DD, Beebe TJ, Davern M, Call KT (2007) Agreement between Self-reported and Administrative Race and Ethnicity Data among Medicaid Enrollees in Minnesota. Health Serv Res 42 (6 Pt 2) 2373–2388.. 17995548 10.1111/j.1475-6773.2007.00771.x PMC2151315

[14] Elliott MN, Fremont A, Morrison PA, Pantoja P, Lurie N, et al (2008) A New Method for Estimating Race/Ethnicity and Associated Disparities Where Administrative Records Lack Self-Reported Race/Ethnicity. Health Serv Res 43 (5 Pt 1) 1722–36.. 18479410 10.1111/j.1475-6773.2008.00854.x PMC2653886

[15] Lauderdale DS, Kestenbaum B (2000) Asian American Ethnic Identification by Surname. Popul Res Policy Rev 19: 283–300..

[16] Goldstein LB, Samsa GP (1997) Reliability of the National Institutes of Health Stroke Scale: Extension to Non-Neurologists in the Context of a Clinical Trial. Stroke 28 (2) 307–310.. 9040680 10.1161/01.str.28.2.307

[17] Muir KW, Weir CJ, Murray GD, Povey C, Lees KR (1996) Comparison of Neurological Scales and Scoring Systems for Acute Stroke Prognosis. Stroke 27 (10) 1817–1820.. 8841337 10.1161/01.str.27.10.1817

[18] Adams H Jr, Bendixen B, Kappelle L, Biller J, Love BB, et al (1993) Classification of Subtype of Acute Ischemic stroke. Definitions for Use in a Multicenter Clinical Trial. TOAST. Trial of Org 10172 in Acute Stroke Treatment. Stroke 24 (1) 35–41.. 7678184 10.1161/01.str.24.1.35

[19] Kolominsky-Rabas PL, Weber M, Gefeller O, Neundoerfer B, Heuschmann PU (2001) Epidemiology of Ischemic Stroke Subtypes According to TOAST Criteria: Incidence, Recurrence, and Long-Term Survival in Ischemic Stroke Subtypes: A Population-Based Study. Stroke 32 (12) 2735–2740.. 11739965 10.1161/hs1201.100209

[20] Association AD (2010) Diagnosis and Classification of Diabetes Mellitus. Diabetes Care 33 (Supplement 1) S62–S69.. 20042775 10.2337/dc10-S062 PMC2797383

[21] JNC-7 Guidelines (2003) The Seventh Report of the Joint National Committee on Prevention, Detection, Evaluation, and Treatment of High Blood Pressure. Hypertension. 10.1161/01.HYP.0000107251.49515.c2 14656957

[22] Diagnosis and Classification of Diabetes Mellitus (2010) Diabetes Care 33 Suppl 1: S62–69.. 20042775 10.2337/dc10-S062 PMC2797383

[23] Current Cigarette Smoking Among U.S. Adults Aged 18 Years and Older. Available: http://www.cdc.gov/tobacco/campaign/tips/resources/data/cigarette-smoking-in-united-states.html#asians

